# Solid Dispersion Formulations by FDM 3D Printing—A Review

**DOI:** 10.3390/pharmaceutics14040690

**Published:** 2022-03-23

**Authors:** Garba M. Khalid, Nashiru Billa

**Affiliations:** 1 Department of Pharmaceutics and Pharmaceutical Technology, Bayero University, Kano P.M.B. 3011, Nigeria; khalidgmk@gmail.com; 2 Pharmaceutical Sciences Department, College of Pharmacy, QU Health, Qatar University, Doha P.O. Box 2713, Qatar

**Keywords:** additive manufacturing, 3D printing, 4D printing, fused deposition modeling, personalized therapy, precision medicine, poorly soluble drugs, solid dispersion

## Abstract

Additive manufacturing (AM) is revolutionizing the way medicines are designed, manufactured, and utilized. Perhaps, AM appears to be ideal for the fit-for-purpose manufacturing of medicines in contrast to the several disadvantages associated with the conventional fit-for-all mass production that accounts for less than 50% of pharmacotherapeutic treatment/management of diseases especially among children and elderly patients, as well as patients with special needs. In this review, we discuss the current trends in the application of additive manufacturing to prepare personalized dosage forms on-demand focusing the attention on the relevance of coupling solid dispersion with FDM 3D printing. Combining the two technologies could offer many advantages such as to improve the solubility, dissolution, and oral bioavailability of poorly soluble drugs in tandem with the concept of precision medicine and personalized dosing and to address the dilemma of commercial availability of FDM filaments loaded with Class II and/or Class IV drugs. However, thermal treatment especially for heat-sensitive drugs, regulatory, and ethical obligations in terms of quality control and quality assurance remain points of concern. Hence, a concerted effort is needed between the scientific community, the pharmaceutical industries, the regulatory agencies, the clinicians and clinical pharmacists, and the end-users to address these concerns.

## 1. Introduction

Conventional mass manufacturing of dosage forms is only 30% successful in achieving intended therapeutic outcomes because it is based on a fit-for-all modality, which does not account for the needs of all patients [1]. This shortfall has prompted intense interests in soliciting more effective treatment modalities tunable to individual patient needs. Such “personalized medicine” provisions recognize that there are significant genetic predispositions amongst patients, of key drug metabolizing enzymes so that patients respond differently to the same medication or dose [2]. Furthermore, chromatin modifications imposed by external factors are triggers for epigenetic aberrations on DNA, which modulate the expression of several phenotypes, including drug-metabolizing enzymes. Thus, the need to tune therapeutic requirements to individual patient needs is ever so relevant [3].

In order to align the desirable attributes of the concept of personalizing medicines to its ultimate application, production methods for prototypes should be intelligent, simple, and quick to fabricate [4]. Unfortunately, conventional manufacturing processes fall short of these attributes, being laborious with multi-station production lines and inflexibility with regard to individualized dose provisions. On the other hand, additive manufacturing (AM) involves the use of materials to print layer-by-layer, 3 dimensional (3D) geometries of dosage forms containing personalized doses based on a digital design [5].

The potential benefits of 3D printing in healthcare is overwhelming as evidenced by the shear scholarly output in the past 10 years [6]. Several 3D printing technologies are applicable to the provisions of personalized medicines. However, fused deposition modelling (FDM) appears to have the most traction in this regard [7]. Furthermore, FDM is one of the more popular 3D printing techniques due to simplicity [8]. Active pharmaceutical ingredient (API)-impregnated thermoplastic polymers casted as filaments by hot-melt extrusion are used as fed material for deployment through the nozzle of the print-head during printing [8]. The physical properties of the 3D printout depend on several parameters that are (i) extruder-related, (ii) process-related, and (iii) structural-related [9]. On the other hand, the release of API from the printout depends on the printing process as well as the physicochemical properties of the API and those of the filament/carrier system. In this context, it is important to note that poorly soluble APIs will likely suffer slow release from the printout, which is compounded by hydrophobicity exhibited by most polymers used in FDM [10]. The application of solid dispersions in conjunction with FDM appears to provide scope for emerging technologies that account for the low solubility of APIs since appropriate polymers can be used to improve the solubility of APIs in solid dispersions. Solid dispersions improve the solubility of APIs by restricting crystal growth. The amorphous state of the API is assured due to intercalation by the solid solvent. Furthermore, solid dispersions improve the dispersibility and wettability of APIs within the solution [11], both of which are crucial precursors to dissolution in solid dosage forms. Polymers such as polyvinyl pyrrolidone [12], polyethylene glycol [13], and hydroxyl propyl methyl cellulose [14] have been successfully used as solid dispersions for improved API solubility. The authors contend that the utilization of solid dispersions in conjunction with FDM in 3D printing presents an expanded frontier in the application of the technology to concepts of personalized medicines, particularly in the context of extemporaneous formulation of poorly soluble drugs on demand at the point of care. In fact, some inroads have already been made recently in this regard [15,16,17] and more scholarly output is envisaged in the near future. Thus, in this review, we appraised the literature on the current trends in the application of FDM coupled with solid dispersions to formulate personalized dosage forms, whereby provision is made for poorly soluble drugs. This is certain to expand the applicability of precision medicine to poorly soluble drugs prepared on small scale. Therefore, the review not only captures the application of solid dispersion in the realm of FDM but also signifies the advantages of this approach with practical examples, challenges, and future perspectives. The literature was generated from Embase^®^ PubMed^®^, and Web of Science^®^ using the following keywords: “additive manufacturing”, “3D printing”, “4D printing”, “fused deposition modeling”, “solid-dispersion”, “personalized therapy”, and “poorly soluble drugs”.

## 2. Additive Manufacturing

Over the last decade, additive manufacturing or three-dimensional (3D) printing technology has been shown to hold a great potential in revolutionizing the designing, manufacturing, and utilization of dosage forms, particularly oral solid and semi-solid dosage forms. Indeed, the approval of the first additive manufactured orodispersible tablet by the United States FDA in 2015 highlights the commitment of the scientific community in conjunction with the pharmaceutical industry towards application of this technology in pharmaceutical sciences. This cooperation underpins the role of pharmaceutical technology in digital health and 21st century innovations in patient management strategies. Aptly, the issuance of the FDA guidelines on additive manufacturing in 2017 paved the way for the growing needs of this technology in the medical, pharmaceutical, and allied fields. The FDA-approved Spritam^®^ tablet was formulated by spraying binder solution on successive layers of powder bed to form a loose 3D compact. The process required use of specialized facilities and is not flexible for small-scale and on-demand manufacturing of dosage forms tailored to specific patient needs [18,19]. This shortfall opened the window for further innovations within this technological framework amenable for small-scale, on-demand, and fit-for-purpose manufacturing of medicines, especially as the recognition of the importance for personalized applications grew. Indeed, a recent survey regarding the implementation of 3D printing technologies in the European pharmaceutical industry primed for personalized medicine highlights the role of community pharmacies and pharmacists, hospital pharmacies, and compounding facilities in the design and preparation of 3D-printed dosage forms for patients use [5].

Technologically speaking, additive manufacturing embodies the use of cost-effective digitally modeled designed (DMD) software to generate different dimensions of a 3D object (solid and/or semi-solid dosage forms) and the application of various automated pharmaceutical engineering techniques for sequentially layering material to construct the pre-designed object. In applied pharmaceutics, additive manufacturing has shown the budding to generate solid [20,21] and semi-solid [22] dosage forms in a rapid, reproducible, and flexible manner [23]. The application of additive manufacturing is advanced in biomedical engineering, construction, automobile, aerospace, fashion, and art industries [24]; however, its application in pharmaceutical engineering only gained momentum during the last decade.

### 2.1. Design of Additive Manufacturing Process

**Figure 1:**
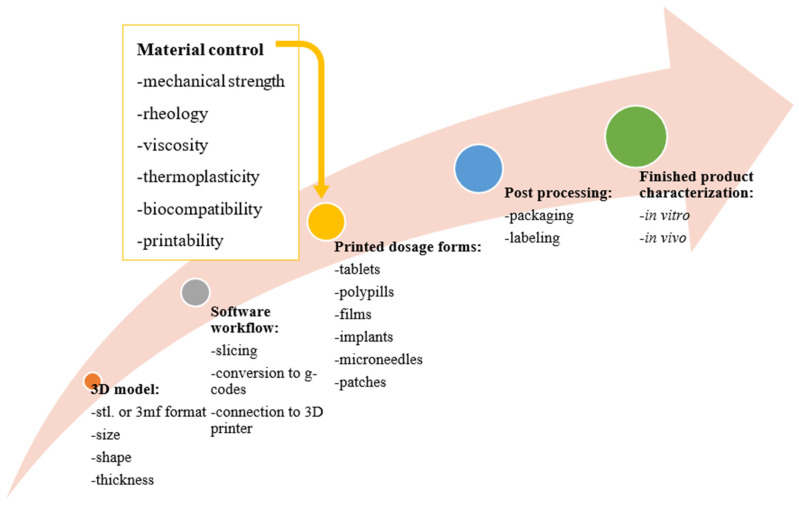
Schematic illustration of the additive manufacturing process for pharmaceutical products, Reproduced from [25], IntechOpen, 2021.

To design and produce pharmaceutical dosage forms by 3D printing, several factors must be taken into consideration, including: (i) the computer-aided design (CAD) software characteristics in relation to the technological platform that will translate the digital design into the 3D object and the type of pharmaceutical dosage form to be generated/formulated (shapes, size, and thickness), (ii) the thermoplastic and rheological behavior of the polymer and other formulation additives, (iii) the type of API to be impregnated and its compatibility/miscibility with the other formulation excipients, and (iv) the effect of the applied technology to the stability of the API. Furthermore, environmental impositions can also affect the quality of the finished product. For equipment without a controlled printing compartment, the ambient conditions can impact the printability and the quality of the printout. Consequently, the FDA guidelines for additive printing enumerates key considerations based on the dimensions, compartment area, and orifice/nozzle type, among others, required for optimized printout [25]. The pre-designed dimensions define the dose of loaded API according to individual patient needs. Once the design is converted to a digital file, the software converts the stereolithographic (STL) file or 3D Manufacturing Format (3DMF) to generate a g-code (computer numerical control programming language) that requires files to be compatible across the different software applications used and the 3D printers. The file is processed for printing by manual controls on the 3D printer (Figure 1). Of relevance to the remit of this review is the nature of the material used for printing, as it relates to suitability for human consumption biocompatibility, toxicology, and biodegradability profiles. After printing is complete, post-processing of the built dosage form (packing and labeling) takes place. Finally, the finished dosage form is ready for in vitro and in vivo characterizations depending on the type of the dosage form [25].

### 2.2. Additive Manufacturing in the Context of Personalized Therapy and On-Demand Patient Care

Additive printing presents a promising premise for the point-of-care manufacturing of medicines. Technologies such as two-dimensional (2D), three-dimensional (3D), and four-dimensional (4D) printing can be used to produce individualized oral drug delivery systems, such as orodispersible films, tablets, and cutaneous drug delivery systems with customizable doses and pre-defined drug release profiles [26,27]. Interestingly, the emergence of 4D printing consolidates the relevance of 3D printing in the context of personalized therapy, precision dosing, site-specific drug delivery, and prolonged retention [28,29]. Four-dimensional printing involves the processing of shape memory materials (SMMs) through 3D printing of smart feed materials that self-transforms in response to external stimuli. Thus, in addition to the x, y, and z-axis for the definition of a shape, 4D printing takes into account the evolution of these coordinates as a function of time [28].

The use of CAD software to design and print 2D, 3D, or 4D drug delivery systems represents a potential of applying additive printing to the concepts of precision dosing of APIs and personalized therapy [27]. Personalized therapy is an emerging therapeutic framework that harnesses different technological innovations to address individual patient needs. Despite significant inroads in conventional pharmaceutical manufacturing in the 21st century, industrial production processes are based on a “one size fit for all” modality, which may be suitable to only a fraction of the population. It does not account for salient genetic predispositions amongst patients. This shortfall is particularly crucial for low and narrow therapeutic-indexed drugs [25]. Additive printing technologies provide an indispensable platform that facilitates the provision of patient-tailored medicines and on-demand [30]. Moreover, this technology is applicable to the printing of medicines in pandemic situations, whereby shortages can be mitigated in a relatively short time [31,32] to reduce cost implications and preparation of individualized fixed-dose combination products that can benefit a myriad of patients [32,33,34].

A key provision in the successful implementation of the concepts of personalized therapy is that it should be applicable to a diverse range of patient groups. This is crucial in pediatric and geriatric patients because of their different/altered physiological and anatomical predispositions to the “normalized” population [35]. Continuous dosage forms such as liquids allow a fair degree of calibration of dose to the needs of specific age groups; however, such provisions are non-existent in solid dosage forms. It is challenging to the pharmaceutical industry to mass-produce all possible solid dose forms for the entire spectrum patient groups because of low profitability. Due to the constraints of using pediatric patients in clinical trials, studies are typically conducted on normalized population groups and the data extrapolated to pediatric patients. There is also a lack of regulatory clarity in the development of pediatric dosage forms [35]. These constraints have led to the emergence of several pediatric dosage forms including mini-tablets, solid dosing pens, colorful sprinkle, granules, dispersible tablets, chewable tablets, chewing gum, and oral gel formulations [35,36]. More recently, the versatility of AM in fabricating solid oral dosage forms, such as orodispersible tablets (ODT) and orodispersible films (ODF), both of which are applicable to pediatric, geriatric, or dysphagic patients has been highlighted [37]. Indeed, on-demand extemporaneous formulation of these dosage forms by AM with different attractive colors and shapes, such as colorful cartoon shapes, candy shapes, or different animal shapes with appropriate palatability, have been shown to improve acceptability and medication adherence in children [38,39,40,41]. Moreover, to address medication errors in the visually impaired, Awad and co-workers designed and prepared orally disintegrating tablets with braille and moon patterns by Selective Laser Sintering (SLS) 3D printing to help such patients identify their medications. This most certainly encourages self-administration of medicines and improves compliance and treatment outcomes [42]. All these highlight the relevance of AM in the context of addressing patient-specific needs to enhance dose accuracy, the availability of medicines on-demand, and achieving overall improved therapeutic goals.

### 2.3. Additive Manufacturing Approaches for Drug Delivery Systems

Several additive manufacturing technologies have emerged, with each technology serving peculiar features to the drug delivery system printed. Some technologies are applicable to a wide spectrum of APIs, whilst others are limited. In general, additive manufacturing technologies can be broadly divided into three categories based on the type of polymeric material used; (i) powder-based techniques, e.g., selective laser sintering (SLS), selective laser melting (SLM), laser metal deposition (SMD), and electron beam melting (EBM); (ii) liquid-based techniques; e.g., stereolithography (SLA), inkjet printing (IJK), and digital light processing (DLP); and (iii) solid-based techniques, e.g., hot-melt extrusion (HME), fused deposition modeling (FDM), electron beam free fabrication (EBFF), and wire and arc additive manufacturing (WAAM) [29]. For the purpose of the present review, we shall focus on FDM technology.

#### 2.3.1. Fused Deposition Modeling Additive Manufacturing

**Figure 2:**
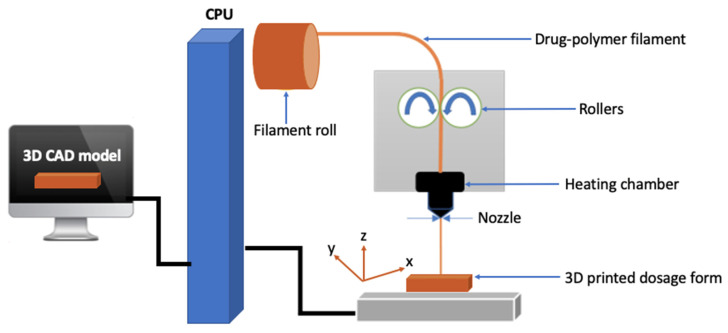
Main features of FDM 3D printing process from computer-aided design to printed dosage form.

Fused deposition modeling (FDM) 3D printing works based on the principles of hot-melt extrusion, and it is well known in the field of pharmaceutical technology dating as early as the late 1990s [43]. It is arguably the most popular and widely applied additive manufacturing technique in dosage form engineering. Indeed, publication in FDM 3D printing has shown geometric progression since 2010 at the expiration of the first patented FDM [43] product. Furthermore, in the last six years, there has been an over 2000-fold increase in the number of published articles related to FDM 3D technology [44]. Thanks to its simplicity, it is applicable to the fabrication of on-demand dose-flexible printouts, involving fewer processing steps and thus is cost-effective [43,45,46]. In this technique, thermoplastic polymer(s) preferably impregnated with API in filaments via hot-melt extrusion is loaded to the nozzle of the printhead, with a specific diameter at a defined temperature. The molten API-loaded filament is deposited on the printing plate layer-by-layer to generate a 3D object based on the CAD. The printhead moves in 3-dimensional (x, y, z) coordinates and successive molten API-loaded filament deposit to the preceding layer (Figure 2). The printing plate, which could also be heated at a specific temperature moves one step lower, or the nozzle moves one step upward to allow for subsequent deposition to occur. The nozzle temperature can be adjusted according to the thermal properties of the API and/or the API-loaded filament as well as the rheological properties of the extrudate [24,43]. Thermoplastic polymers commonly used in FDM include acrylonitrile butadiene styrene (ABS), polycarbonate (PC), polylactic acid (PLA), polystyrene (PS), polyamide (PA), polyether ether ketone (PEEK), and lignin [47,48]. However, not all are suitable for 3D FDM fabrication of pharmaceutical dosage forms for human consumption due to safety concerns.

Due to the proliferation of FDM in pharmaceuticals and allied medical fields in the last decade, the HME technology is grossly employed in the production of feedstock material for FDM 3D printers comprising of thermoplastic polymers in the form of filaments with diameters ranging between 1.75–3.00 mm. This provision requires that process parameters and polymer characteristics be carefully calibrated to produce filaments with desirable characteristics for specific applications [43].

##### Material Requirements for FDM AM

**Figure 3:**
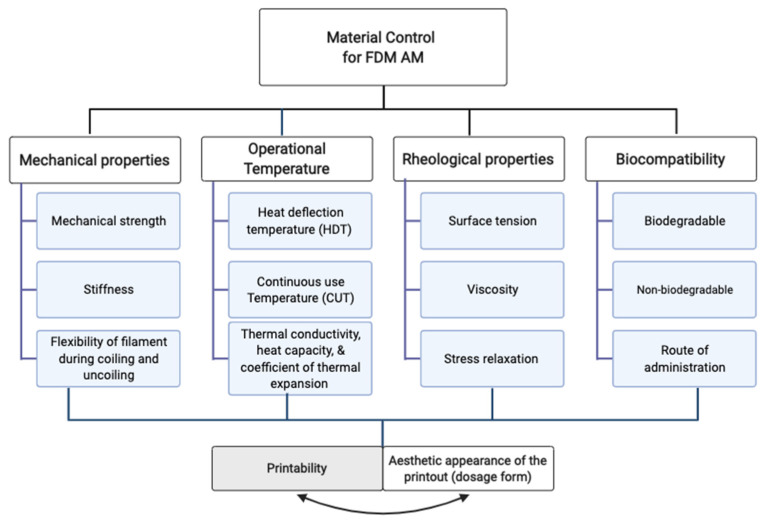
Schematic illustration of material control for FDM additive manufacturing.

The choice of the right material for a successful FDM 3D printing is crucial to the printing process once the FDM 3D printer design is selected. Although the environmental conditions such as ambient temperature and relative humidity have been shown to significantly influence the material behaviors during and after 3D printing [20], the intrinsic property of the material is more critical since the environmental condition can be controlled. Mechanical strength, operational temperature, rheological properties, biocompatibility, and biodegradability are some of the critical quality attributes to consider when selecting a thermoplastic material for fabrication using FDM 3D printing technology (Figure 3) [49].

*Mechanical strength*: In FDM printing, the solid filament used acts as a piston under compression during the extrusion process; therefore, it should retain sufficient mechanical strength to prevent buckling between the feed pinch roller and the heated liquefier. On the other hand, it also needs to be flexible enough to enable coiling and uncoiling during production and the printing process, respectively [50]. Thus, an ideal filament should have a minimum strain at a yield of about 5% [51,52]. Filaments with a high Young’s modulus (>300 N/mm^2^) can be conveyed without buckling or deforming [53,54]. For more details on the most commonly used thermoplastic polymers in FDM AM and application of simple screening method to assess mechanical qualities and printability, the reader may kindly refer to the work of Nasereddin and co-workers [55].

*Processing temperature*: The Heat Deflection Temperature (HDT) is the temperature at which materials deform plastically under load bends, whilst the Continuous Use Temperature (CUT) indicates the temperature at which materials can work continuously for an indefinite time [49]. The former is related to a lag time of heating required to initiate liquefication of the filament prior to extrusion, whilst the latter is related the temperature maintained during the extrusion and printing. Both are crucial to the choice of the material because, often, the mechanical resistance of the printout is a function of the operational temperature. Thus, it is important to select a material that is stable after exposure to both HDT and CUT. During the FDM AM process, printouts often undergo shrinkage upon cooling, which is affected by the thermal properties of the filament, including thermal conductivity, heat capacity, coefficient of thermal expansion, and/or crystallinity in the case of semi-crystalline filaments. Uneven shrinkage often happens because of thermal gradients between the successive printouts, which may lead to warpage and even delamination or cracking of the printouts [50,52]. However, amorphous filaments have a high dimensional accuracy and are thus preferred in FDM AM, while semi-crystalline filaments offer better mechanical properties but may influence higher shrinkage and warpage. Therefore, most FDM printers are equipped with a heating element attached to the printing plate to minimize the temperature differential between successive printout layers [52].

*Rheological properties*: As a result of sequential layer-by-layer deposition during FDM AM, intralayer adhesion and interlayer adhesion occur when the new extrudate is deposited beside and on top of the initial layout, respectively. The fusion of the interface allows intra-layer or interlayer adhesion between two extrudates to occur via a time-dependent mechanism that involves viscous flow and polymer chain diffusion. Such fusion is influenced by the rheological properties of the polymer including its surface tension, viscosity, and stress relaxation behavior upon deposition [56,57]. Moreover, it has also been observed that even distribution of a suitable filler in the polymer matrix can alter the rheological behaviors of the filament and improve the stable filament diameter, and filament strain at yield, thus facilitating processability and stability of the printout during and after 3D printing, respectively [51,58,59].

*Biocompatibility and biodegradability*: Depending on the area of application of the dosage form fabricated through FDM AM, it is desirable for the polymeric material to be biocompatible and/or biodegradable. For dosage forms destined for used in vivo, both attributes are required in most cases. For dosage forms designed for application in body cavities or for topical applications, the polymer may ideally be biocompatible but not necessarily biodegradable. Biocompatible polymers have the capacity to release the API(s) from a dosage form without causing any significant changes in the physiological functions of the body or the body fluids. On the other hand, biodegradable polymers decompose naturally in a bioactive environment by microorganisms such as bacteria and fungi [60]. Moreover, the long chains of polymers can break down into monomers due to the enzymatic actions of microorganisms [61]. Bio-based thermoplastic polymers are derived from renewable biomass sources such as starch, vegetable fats and oils, straw, and woodchips. It is therefore essential to explore different thermoplastic bio-based materials for FDM filaments to advance the range of naturally derived filament materials [62].

Other material requirements are printability, the aesthetics of the printout, and the cost [49]. The printability has to do with the reproducibility of the printout and its consistent quality, while the aesthetic appearance of the printout and cost can significantly influence patients’ compliance to FDM-printed medicines and ultimately the therapeutic success of the medication. Thus, these variables are directly related to both the FDM 3D printer operator/expert and the patients.

##### FDM Filament Characterization

Filament evaluation is very important step in optimizing the quality of the filament because it eventually defines the quality of the finished pharmaceutical dosage form fabricated by FDM AM. Various analytical techniques can be applied both off-line and in-process. For the former, physical evaluation of the placebo and drug-loaded filaments will give information regarding potential changes in color or texture due to thermal degradation of APIs or some of the excipients used. The filament is also required to have sufficient mechanical strength for efficient printing without deformities as discussed in Section ‘Material Requirements for FDM AM’ under ‘mechanical strength’. Other filament parameters to be evaluated off-line include the water content and the water sorption capacity of the filament. Water sorption during processing or storage can act as plasticizer whereby the glass transition temperature of the filament is lowered. Moreover, this does not only affect the physical appearance and mechanical properties of the filament but can also potentiate microbial growth, altering drug stability and eventually the stability of the dosage form [54,63]. The drug release pattern in vitro can be assessed using relevant pharmacopeial specifications depending on the type of the API loaded. The analytical HPLC method is used to evaluate the drug content, the uniformity of the API distribution within the filament, and to detect and quantify the presence of potential degradation products [54,64]. In most cases, these tests are also applicable to the final dosage form(s).

Non-destructive in-process analytical techniques such as rheometry, optical coherence tomography (OCT), and spectroscopy can be utilized to obtain real-time information regarding the process and filament properties during filament preparation. Filament rheological properties such as the influence of API on the torque can be assessed by in-process rheometry [65], while OCT can be used to evaluate surface properties of filament, layer thickness, diameter, and sphericity of the filament [66,67]. Spectroscopy such as inline near infrared (NIR) spectroscopy can be used to investigate drug–polymer interactions and to validate a method for continuous API quantification during filament processing [68].

Solid-state characterization is another in-process filament quality assessment measure. Filament loaded with poorly soluble APIs can be prepared as amorphous solid dispersions to enhance drug solubility where the crystalline structure of the API is broken up and the resulting molecular dispersions are stabilized within a network of polymer matrix. The solid-state of an API incorporated in a filament for FDM AM can have an influence on the biopharmaceutical performance of the final dosage form with respect to solubility, the dissolution rate, absorption, and bioavailability [69]. Analytical techniques such as X-ray powder diffraction (XRPD), differential scanning calorimetry (DSC), and polarized light microscopy [70] are among the well-established methods to assess the solid-state of API dispersed in polymer matrices. Although, the later lacks selectivity and quantitative wit. Thus, it is of importance to evaluate the solid-state properties of the filament prior to printing by FDM.

##### In-Process Quality Control for FDM AM

There are two main critical quality control remits ascribable to FDM additive manufacturing, namely, the machine parameters (printing plate/bed calibration and nozzle diameter control/selection,) and the process parameters (nozzle temperature, printing plate/bed temperature, extrusion width, and raster/printing angle). The printing plate calibration is a crucial consideration in FDM 3D printing since the distance between the nozzle and printing plate needs to be synchronized and remains constant throughout the plate space. Improper plate calibration may lead to uneven deposition of the extrudate and messy resolution of the printout. The diameter of the 3D printing nozzle dictates the rate of the extrusion of the melt material [71]. Nozzle temperature has been reported to significantly influence the mechanical properties and microstructure of the 3D-printed dosage form [71,72]. The raster angle of about 45° favors high-quality printouts, while other angles may bring about messy printing [73]. An optimal plate temperature is required to impart adhesion between the printing plate and the 3D object and avoid distortion [29,74]. The nozzle speed influences the dimensional quality and precision of the printout such that at high speed, the quality of the printout is reduced [29,75] through uneven diameter, poor mechanical properties, and the inclusion of bubbles [45,55,76]. Therefore, for successful FDM-based additive manufacturing, the key is the proper selection of FDM printer and process parameters optimization. Appropriate parameters can improve the printing process, the quality of the printout, and subsequent functionalities of the dosage form [73].

As mentioned earlier, the main advantages of FDM additive manufacturing in pharmaceutical sciences include the availability of a wide range of thermoplastic materials adaptable for the technique, the ability to print customizable dosage forms with different fillings and dimensions within a short period, and low costs compared to other additive manufacturing techniques [77].

##### Limitations of FDM 3D Printing

Despite the possible applications of FDM AM in personalized therapy, the technology has some inherent limitations in drug-specific fabrication of delivery systems. These limitations include the sequential exposure of the drug-polymer and API-loaded filament to high temperatures during hot-melt extrusion and FDM 3D printing, respectively. This may induce API degradation and thus limit the scope of drugs applicable to this technology. Furthermore, the dissolution profile of API from the 3D printout is often impeded due to the plastic nature of the printout. Thus, for low-solubility APIs, a further constraint is imposed. The application of FDM to the extemporaneously prepared dosage forms such as ODT or ODF is complicated by the fact that drug-loaded filaments have to be initially formed by hot-melt extrusion (HME) or obtained by impregnation of commercial filament [21]. The former requires double exposure of the drug and polymer material to extreme temperatures in HME and FDM 3D printing with a consequence of provoking thermal degradation of the APIs and/or other formulation excipients [78]. In addition, HME often generates brittle or inflexible filaments that cannot be loaded to an FDM 3D printer [79]. Impregnation of the filament with API(s) may result in weakening the filament and lowering its suitability for onward printing and/or handling of the printout by the patients during administration [7]. The lack of commercially available drug-loaded filaments for FDM 3D printing is another constraint in the application of this technology. Hence, there is a need to continue optimizing printing processes in order to achieve dose precision and printing quality [80]. It is the opinion of the authors that, by addressing these hurdles through research and development, it is possible to extend this technology in personalized dosing. We propose further research, leveraging on the principles of solid dispersions, in FDM 3D printing to account for the low solubility APIs as well as potentially minimizing high melt temperatures so that a wider scope of API candidates can be considered in FDM AM technology.

##### Scalability of FDM 3D Printing

FDM 3D printing has potential uses as a digitized tool for personalized dispensing of drugs. However, unlike other AM technologies such as powder bed 3D printing, which was used to produce the first commercial 3D printed dosage form, the scalability of FMD 3D printing to industrial mass production may face several hurdles due to the necessity to increase the speed of printing. As elaborated in Section ‘In-Process Quality Control for FDM AM’, high-speed printing results in poorly formed printouts. However, it may be possible to construct more than one nozzle to produce several printouts simultaneously. This would require the inclusion of a large printing platform. Scale-up production will also require long drug-impregnated filament spools to permit uninterrupted printing. Although there is value in scaling up AM processes, where dose personalization is required, the economies of scaling up of FDM 3D printing will likely never reach the same level as conventional mass production [50]. However, there is scope for partnership between the pharmaceutical industry and compounding pharmacies to scale-up FDM 3D printing. For example, the pharmaceutical industry can mass-produce drug-impregnated filaments with key quality and safety specifications for use in hospital or community pharmacy settings. This will afford the requirements of personalized medicines with scope to scale-up production by FDM downstream [81]. Moreover, adaptations to current commercial FDM 3D printers will need to meet regulatory requirements of GMP [54].

## 3. Solid Dispersions

Various techniques have been employed to enhance the solubility, dissolution rates, and ultimately the oral bioavailability of poorly soluble APIs, among which include solid dispersion [82,83], pH modification, crystal modifications (co-crystals, polymorphs, and salts), amorphization, surfactant systems, particle size engineering, cyclodextrin complexation, and lipid-based formulations [83,84,85,86,87]. Solid dispersion is feasible, easy to formulate, economical, and has been extensively employed in this regard [83,85,87]. Solid dispersions date back to 1961 [88] and have been refined over the years with current application on hydrophobic APIs, typically Class II and IV drugs of the Biopharmaceutics Classification System (BCS). The resultant solid dispersed system accounts for improved solubility of the API through intercalation of the crystalline domains with dispersant that transforms it to an amorphous or mono-molecularly dispersed state [83,89]. Furthermore, enhanced wettability, dispersibility, and reduced aggregation of API augments the process of solution [89]. Inclusion of surfactants can further improve solubility [83].

### Mechanism of Solid Dispersion in Context of Drug-Polymer Interaction

**Figure 4:**
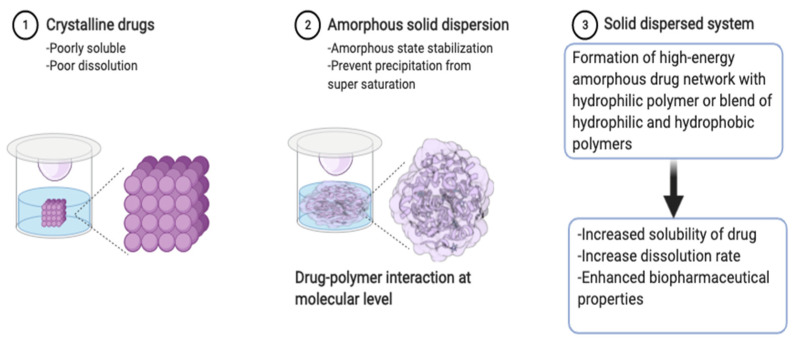
Schematic illustration of solid dispersion process.

Solid dispersions are eutectic mixtures or solid solutions in which API exist either in an amorphous form dispersed in the carrier or as a molecular dispersion in the carrier to enhance the drug supersaturation level while preventing drug crystallization [90,91]. Indeed, the formation of a high-energy amorphous form or increased solubility due to supersaturation in solid dispersion favor enhanced dissolution of hydrophobic drugs. The increased solubility can be attributed to the dispersion of drugs at the molecular level and/or solubilization effects of the polymer. The API remains in a metastable form for a considerable time in the supersaturated state, and the polymeric carrier in turn can stabilize the metastable state by preventing nucleation and subsequent crystallization as highlighted in Figure 4. Polymers that strongly inhibit the crystallization of an API can maintain supersaturation [92]. Hence, the API/polymer ratio in a solid dispersion affects the drug dissolution rate from the solid dispersion and may influence the dissolution mechanism of the API from such systems [91]. For example, when the API content is relatively low, it dissolves simultaneously with the hydrophilic polymer from the solid dispersion, and dissolution can be controlled by the characteristics of the polymer carrier. For a solid dispersion with carrier-controlled dissolution, the use of a fast-dissolving polymer as a carrier is an effective method to enhance drug dissolution [93,94]. Aptly, these features cannot be achievable by one single polymer. Perhaps, the polymer dissolution rate itself can be enhanced by polymer blending, which modifies the molecular interaction between the hydrophobic API and the polymer blend [95]. Thus, the molecular interaction between the API and each polymer in the ternary solid dispersion is an important factor in defining the API dissolution properties [96].

The methods used to prepare solid dispersions could affect the molecular state and dissolution characteristics of an API [92]. In addition, a suitable choice of polymer is essential for a stable solid dispersed system. API crystallization from the supersaturated solution should be efficiently suppressed in the presence of the polymer. Furthermore, the polymer structure, such as the functional groups, has been reported to affect the preservation of the supersaturated state of hydrophobic APIs in solid dispersion [97]. Consequently, some studies have reported the merits of blending hydrophobic and hydrophilic polymers in ternary solid dispersions by promoting optimized dissolution rate and supersaturation maintenance relative to the respective binary solid dispersed systems [95,96,97,98]. 

Solid dispersions can be fabricated by HME, melting/fusion [99,100,101,102], or solvent evaporation after co-precipitation, by supercritical fluid, lyophilization, and electrospinning [88,103]. Melt solvent or melt evaporation and kneading [89] are also applicable methodologies. Solid dispersants commonly employed include polyvinylpyrrolidone (PVP), polyethylene glycol (PEG), hydroxypropyl cellulose (HPC), hydroxypropyl methylcellulose acetyl succinate (HMPC-AS), and hydroxypropyl methylcellulose (HPMC). Careful selection of dispersants in appropriate amounts have been shown to enhance the solubility of drugs. For example, the dissolution of griseofulvin from solid dispersions of PVP was improved 11-fold in a 1:20 drug/carrier ratio [100]. The miscibility and kinetic stability of miconazole solid dispersion was improved through hot-melt extrusion using a copolymer graft of polyethyleneglycol-g-vinyl alcohol [104]. The huge potential that solid dispersions holds in improving the solubility of API is evident by the number of such products that have emerged in the market, for example, Fenofibrate (Fenoglide^®^), Duloxetine (Cymbalta^®^), and Vemurafenib (Zelboraf^®^), in different dosage forms cannot be overemphasized. We will now discuss further potential of solid dispersion technology when used in conjunction with FDM.

## 4. Solid Dispersion Coupled with FDM Additive Manufacturing

Application of FDM in conjunction with solid dispersion technology is based on the premise that both technologies essentially rely on an API–polymer admixture. From the discussions of the key provisions of each technology, it is conceivable that a coupled technology brings together the advantages of the two approaches as a single system. Indeed, solid dispersion technology is currently being explored to preliminarily obtain API-loaded filament for onward use in FDM additive manufacturing [15,39]. Even though this coupled technology has not received the attention it deserves, the merits associated with it are very palpable. This coupled technology has the potential of expanding the portfolio of APIs akin to FDM.

### 4.1. Advantages of Coupling Solid Dispersion with FDM Additive Manufacturing

In-house preparation of FDM API-loaded filaments by solid dispersion techniques will address some of the limitations associated with FDM 3D printing. First, the commercial dilemma of API-loaded filaments is currently the main constraint to actualizing the full potential of FDM additive manufacturing in personalized dosing, especially for BCS class II and class IV drugs. Filaments with optimum mechanical properties, flexibility, thermal stability, and melt viscosity to pass through the printer nozzle are key requisites. Yet, most polymers used in the development of pharmaceutical dosage forms lack such qualities because extruded filaments are either too brittle and break during or after the 3D printing. Filaments may also be too soft to be pushed by the FDM printer’s drive gear [105,106]. The addition of fillers and plasticizers may address some of the negative characteristic but leads to the design of complex formulations, adds to the weight of the dosage form, and has the potential of impeding the stability of the of the printout [47]. Therefore, it is the authors’ view that in-house-made drug-loaded filament can address these challenges and make it easier to generate customizable filaments with improved stability especially when such filaments are needed in small quantities. This can provide an added value in the context of pharmacoeconomics to both the formulation experts and the patients since the former does not need to order large consignments of commercial rolls of the drug-loaded filament(s), which can amount to a high cost and which might not be available. Secondly, the majority of APIs currently available in the market or emerging from development pipelines are poorly soluble and or display pH-dependent solubility. Consequently, the release of such APIs from FDM 3D-printed dosage is faced with solubility-dependent rate processes. Such drugs are best presented in the amorphous state as solid dispersed systems so that release is dissolution, erosion, or dispersion controlled rather than solubility-rate controlled [47]. Furthermore, the majority oral dosage forms currently on the market are immediate-release tablets [107]. Hence, solid dispersion coupled with FDM additive manufacturing provides some leverage to address the above limitations. Both solid dispersion and FDM 3D printing can be materialized without the need for a solvent. Hence, coupling the two in a single drug formulation process can provide scope for the inclusion of more APIs without a significant impact on the FDM process. This ideal in FDM 3D printing will potentiate the fit-for-purpose concept in dose precision and personalized therapy. Third, there is also scope for API delivery with specific features, such as targeted drug delivery. Recently, Melocchi and co-workers demonstrated the efficiency of HME-filament-generated solid dispersion coupled to FDM additive manufacturing to prepare chronotopic drug-delivery systems. This approach assesses the feasibility of fabricating chronotopic systems loaded with caffeine for both pulsatile release and colonic-targeted release configurations based on a time-dependent approach consistent with the needs of personalized medicine [66]. Similarly, filaments composed of haloperidol and polymer carriers; Kollidon^®^ VA64, Kollicoat^®^ IR, Affinsiol™15 cP, and HPMC-AS either individually or as binary blends in solid dispersion by hot-melt extrusion, whereby Kollidon^®^ VA64 and Affinisol^®^15 cP in the ratio 1:1 was identified as most suitable for FDM, exhibiting rapid drug-release profiles [47].

### 4.2. Clinical Potential of Combining Solid Dispersion with FDM 3D Printing

About 40% of approved marketed drugs and nearly 90% of molecules in the drug-discovery pipelines are lipophilic and consequently poorly soluble [90]. To overcome the constraint to formulating these poorly soluble drugs, in viable dosage forms, solid-dispersion-coupled FDM 3D printing can be utilized as economical leverage whereby there is a potential to reduce the cost for drug discovery process for solid dosage forms in development pipelines through hastening pre-formulations procedures. This approach can also facilitate the clinical trials of solid dosage forms loaded with poorly soluble drugs by making it cost effective and achievable with a short runtime due to the rapidity of FDM 3D printing. Extension of the product portfolio of the approved marketed APIs, which are soluble, with a view to improve their efficacy and safety is also a possibility.

Finally, solid dispersion being an excellent technique of increasing the dissolution of poorly soluble drugs [108] provides a premise for combination with FDM 3D printing, imbibing a myriad of clinical benefits such as personalized dose titration to obtain the desired dose strength, reducing polypharmacy and the multiple pill burden, with the ability to use modifiable excipients based on patient peculiarities and clinical condition and the ease of small scale manufacturing and accessibility to medicines on demand in times of disaster such as coronavirus disease 2019 (COVID-19) due to supply chain disruption [109]. Indeed, due to its portability (small size and convenience for application in clinical pharmacy settings), short processing time, and rapid turnover for dosage form production, FDM 3D printing coupled with solid dispersion can be adapted to improve medication compliance by reducing the patient waiting time during extemporaneous compounding in addition to customizing their medicines at the point of use.

HME appears to be the most consequential technique in producing solid dispersions because it offers a one-step, continuous, and solvent-free manufacturing modality and has been the method of choice for use in FDM-based additive manufacturing [45]. Examples of other techniques used in fabricating solid dispersion-based filaments for FDM additive manufacturing with their merits and/or special features are highlighted in Table 1.

## 5. Challenges and Limitations

Despite the several merits outlined earlier that are associated with coupling solid dispersion with FDM additive manufacturing, the fundamental limitation is that APIs recruited should still possess a fairly high melting temperature, which affects the quality of the filament [113,114,115].

Secondly, in most solid dispersion coupled FDM 3D printing studies, the miscibility of API with additives in the polymers used is not investigated. Since the solid dispersion will likely contain additive(s) the miscibility of API of the various components must be studied as a function of thermal exposure. In the absence of such miscibility studies, there is a likelihood of crystallization of the API out in the final dosage form during shelf life, resulting in physical instability and variability in drug release patterns [47,116]. The formulation of filaments by hot-melt extrusion and the 3D printing by FDM involves high temperature, which might transform the API.

Thirdly, due to their thermodynamic instability, solid dispersion products are sensitive to temperature and humidity fluctuations during processing and storage. Thus, these factors must be appropriately controlled [89].

Fourth, simultaneous development of formulation and processing parameters for solid dispersion and FDM 3D printing is challenging because one must satisfy the requirements of high-quality solid dispersion production and filament printability by FDM 3D printing. In particular, the best choices of polymers for given drugs and processing temperatures to generate and maintain the solid dispersion form [47] may not necessarily be acceptable for filament printability [45].

Fifth, regulatory and ethical requirements in terms of quality assurance and quality control in relation to the expected functionality and performance of the different types of the dosage forms (site-specific delivery, prolonged/sustained drug release, and fit-for-purpose personalized dosing) still remains debatable [5,44,54].

Therefore, for successful formulation of solid dispersed FDM 3D-printed dosage forms, polymers must be pharmaceutical grade, the API should be miscible with the polymer, the extruded filaments should be flexible and usable in the FDM 3D printing, and the printing should ideally not require very high temperature [46].

## 6. Conclusions and Future Directions

From the forgoing, it is apparent that additive manufacturing within the realm of fit-for-purpose design and personalization of medicines holds a huge potential. It is also clear that there is room for innovative approaches especially with respect to newer technologies that account for poor API solubility. This expansion in scope adds to the broader requirements of on-demand manufacturing and dose personalization. Indeed, the versatility of FDM 3D printing coupled with solid dispersion appears to hold the key to some of these provisions. On the other hand, pharmaceutical, regulatory, and ethical constraints remain, but are not insurmountable. The sheer number of scholarly outputs employing FDM in conjunction with solid dispersion for 3D printing of dosage forms attests to the potential of this approach. It is the view of the authors that significant inroads based on coupling of the two technologies will emerge with consequential implications to the provisions of personalized medicines.

## Figures and Tables

**Table 1:** Some examples of solid dispersion technologies coupled FDM additive manufacturing and their various applications in personalized dosing.

Solid Dispersion Filament Generated Technology	FDM Printer	FDM Printing Temperature	Dosage Form	Model Drug(s)	Polymer/Excipients	Special Features and Merits	References
HME at 160–190 °C	Kloner3D 240^®^ Twin (Kloner3D, Florence, Italy)	180–195 °C nozzle and 50–100 °C plate for HPC and EDR shells, respectively	Multiple-component reservoir systems in form of shells followed by manual insertion of printed core.	Caffeine	HPC, HPC SSL, Eudragit^®^ L 100-55, PVA, glycerol, PEG 400, TEC, sodium starch glycolate (Explotab^®^), AMY (Amylo^®^ N-460)	Chronotropic system for pulsatile release and colonic targeted delivery. Drug-containing core surrounded by a coat made of swellable/soluble hydrophilic polymers. The swellable polymers provide a programmable lag phase prior to drug release. Manual insertion of previously printed parts (i.e., either the drug-containing core or the two-component pulsatile-release system) into the shell structure under fabrication Precision medicine and dose personalization	[101]
HME at 165 °C (co-rotating twin-screw Extruder, Thermo Fisher Scientific Inc., Waltham, MA, USA)	Flashforge, Creator Pro 3D, 2016, Jinhua, Zhejiang, China	165–240 °C	Tablets	Griseofulvin	HPC, SL grade, Kollicoat^®^ Protect, aqueous SDS	Single-step fusion-assisted amorphization during FDM 3D printing of the crystalline drug. High relative surface area of the drug in the printed tablets. Supersaturation of the drug ~153%. Square-pattern perforated cylindrical tablets with enhanced dissolution rates.	[45]
Solvent evaporation at 60 °C	MakerBot Replicator 2X (MakerBot Inc., Brooklyn, New York, NY, USA)	230 °C nozzle and 20 °C printing plate	Tablets	Fluorescein sodium (FS) and 5-aminosalicyclic acid (5-ASA)	PVA placebo filament, ethanol, methanol, and DMSO	Fixed-dose combination product to reduce polypharmacy. Improvement of filament drug loading by one- to threefold Favorable release profiles of the two drugs	[15]
HME at 90–100 °C (counter flow twin-screw hot melt extruder, HAAKE MiniCTW, Karlsruhe, Germany).	MakerWare Version 3.9.1.1143 (Makerbot Industries, LLC., Brooklyn, New York, NY, USA).	135 °C nozzle and 60 °C printing plate	Tablets	Captopril, Theophylline, prednisolone, 5-ASA	Eudragit EPO, thermally stable filler, TCP, directly compressible lactose Ludipress^®^, spray-dried lactose, MCC, TEC, and talc.	Addition of non-melting filler (TCP) to methacrylic matrix to facilitate reproducible FDM 3D printing Utilized one optimized filament to accommodate four model drugs with different melting points. Fabrication of patient-tailored immediate release tablets of poorly water-soluble drugs	[18]
HME at 150 °C (Process 11 co-rotating twin screw Extruder, Thermo Scientific, Bridgewater, NJ, USA)	MakerBot Replicator 2 desktop 3D printer (MakerBot, Brooklyn, New York, NY, USA)	210 °C nozzle, printing plate at room temperature	Tablets	Haloperidol	Kollidon^®^ VA64, Kollicoat^®^ IR, Affinsiol™15 cP and HPMC-AS either individually or as binary blends	Drug-polymer miscibility study by film casting method using differential scanning calorimetry (DSC), powder X-ray diffraction (PXRD) and polarized light microscopy (PLM) to select the drug-compatible polymer. Rapid dissolution rates and fast drug release	[47]
HME at 140–200 °C (11 Parallel Twin-Screw Extruder, Thermo Fisher Scientific, Waltham, Massachusetts, USA)	Prusa i3 3D desktop printer, Prusa Research, Prague, Czech Republic	200 °C nozzle and 50 °C printing plate	Tablets	Acetaminophen	AquaSolve™, HPMCAS, Benecel™, HPMC E5 and K100M, HPC, Aqualon™ ethylcellulose EC N14	Relevant of combining HME and 3D printing technology as a potential continuous process for personalized dosage development and modified drug release profiles. Miscibility of cellulose-based polymers with the model drug in amorphous solid dispersion	[105]
HME at 140–180 °C (co-rotating, twin-screw extruder, Thermo Fisher Scientific, Waltham, MA, USA)	Prusa i3 3D desktop printer, Prusa Research, Prague, Czech Republic	200 °C nozzle and 50 °C printing plate	Tablets	Acetaminophen	Eudragit^®^ L100, Benecel™ HPMC E5, Klucel™ HPC EF and LF, and Aqualon™ EC N14, Soluplus^®^	Extended/controlled tablets with better release profiles compared to directly compressed tablets Patient-tailored dosing and on-demand manufacturing of medicine	[106]
HME at 130 °C (single screw extruder Noztek Pro, Noztek, Sussex, UK)	Raise3D Pro2, Raise3D Technologies, Inc, Irvine, CA, USA	190 °C nozzle and 70 °C printing plate	Tablets	Theophylline	Polyurethane (Tecoflex™ EG-72D TPU)	Fractal Dimension analysis has been employed for the first time as a non-destructive, non-expensive and fast method for estimating filament printability by FDM 3D printing. High drug-loaded filaments made of polyurethane and anhydrous theophylline (10–70% *w/w* of drug content)	[110]
HME at 150–160 °C (single-screw filament extruder, Noztec Pro hot melt extruder, Noztec, UK)	Ultimaker 3 FDM printer (Ultimaker, Wormer, The Netherlands)	180–190 °C nozzle and 80 °C printing plate	Tablets	Amlodipine	PVA, SSG, Affinisol™ HPMC HME 4 M	Role of excipients selection and/or adjusting the infill pattern and wall thickness as ways of tailoring drug release in FDM 3D printing. High release profiles of the model drug. Different release behavior according to the composition of the tablet and parameters chosen for printing.	[111]
Solvent evaporation and HME (Noztek^®^ Pro filament extruder, Shoreham, England) at 70 °C and 172 °C respectively	ZMorph^®^ 2.0S personal fabricator (Wroclaw, Poland)	185–190 °C nozzle	Orodispersible films	Aripiprazole	PVA, ethanol,	Amorphization of the aripiprazole and porous structure of printed film led to increased dissolution rate.	[77]
Solvent evaporation at 80 °C	In-house modified FDM printer with FDM extruder replaced by linear syringe pump.	70 °C printing plate.	Orodispersible films	Benzydamine hydrochloride	Maltodextrin (Glucidex 6–G6), Sorbitol, HEC (WP 40, QP 300 and QP 4400H)	Modified printing method shows great promise in a compounding of personalized film dosage forms. Preparation of films with compartmented drugs and incorporation of taste masking or release control layers. Control of the dose by changing the thickness & overall volume of digital model	[112]

HME: Hot melt extrusion, HPC: Hydroxypropyl cellulose, HPC SSL: low viscosity Hydroxypropyl cellulose, PVA: polyvinyl alcohol, PEG: polyethylene glycol, TEC: triethyl, AMY: high-amylose maize starch citrate, SDS: sodium dodecyl sulfate, DMSO: Dimethyl Sulfoxide, TEC: Triethyl citrate, TCP: tri-Calcium phosphate, 5-ASA: 5-Aminosalicylic acid, MCC: microcrystalline cellulose, HPMCAS: hypromellose acetate succinate, SSG: Sodium starch glycolate.

## Data Availability

Not applicable.
